# P2X7 Receptor Suppression Preserves Blood-Brain Barrier through Inhibiting RhoA Activation after Experimental Intracerebral Hemorrhage in Rats

**DOI:** 10.1038/srep23286

**Published:** 2016-03-16

**Authors:** Hengli Zhao, Xuan Zhang, Zhiqiang Dai, Yang Feng, Qiang Li, John H. Zhang, Xin Liu, Yujie Chen, Hua Feng

**Affiliations:** 1 Department of Neurosurgery, Southwest Hospital, Third Military Medical University, Chongqing, China; 2 Department of Anesthesiology, Neurosurgery and Physiology, Loma Linda University, California, USA

## Abstract

Blockading P2X7 receptor(P2X7R) provides neuroprotection toward various neurological disorders, including stroke, traumatic brain injury, and subarachnoid hemorrhage. However, whether and how P2X7 receptor suppression protects blood-brain barrier(BBB) after intracerebral hemorrhage(ICH) remains unexplored. In present study, intrastriatal autologous-blood injection was used to mimic ICH in rats. Selective P2X7R inhibitor A438079, P2X7R agonist BzATP, and P2X7R siRNA were administrated to evaluate the effects of P2X7R suppression. Selective RhoA inhibitor C3 transferase was administered to clarify the involvement of RhoA. Post-assessments, including neurological deficits, Fluoro-Jade C staining, brain edema, Evans blue extravasation and fluorescence, western blot, RhoA activity assay and immunohistochemistry were performed. Then the key results were verified in collagenase induced ICH model. We found that endogenous P2X7R increased at 3 hrs after ICH with peak at 24 hrs, then returned to normal at 72 hrs after ICH. Enhanced immunoreactivity was observed on the neurovascular structure around hematoma at 24 hrs after ICH, along with perivascular astrocytes and endothelial cells. Both A438079 and P2X7R siRNA alleviated neurological deficits, brain edema, and BBB disruption after ICH, in association with RhoA activation and down-regulated endothelial junction proteins. However, BzATP abolished those effects. In addition, C3 transferase reduced brain injury and increased endothelial junction proteins’ expression after ICH. These data indicated P2X7R suppression could preserve BBB integrity after ICH through inhibiting RhoA activation.

Spontaneous intracerebral hemorrhage (ICH) is a common fatal stroke subtype due to small vessel bleed within brain parenchyma and subsequent hematoma^1^. Blood–brain barrier disruption is one of the most important pathophysiological changes in early brain injury after ICH and contributes to vasogenic brain edema formation, which leads to poor prognosis^2^,^3^,^4^. Endothelial tight junction proteins, such as Occludin and ZO-1, and endothelial adherent proteins like VE-Cadherin, are important to blood-brain barrier (BBB) integrity^5^,^6^. Decreased expression and disarrangement of the endothelial junction proteins after ICH indicate blood brain barrier integrity disruption and increased paracellular permeability^6^,^7^. Therefore, to protect the blood brain barrier via targeting endothelial junction proteins seems desperately needed and was considered a major promising strategy for the treatment of early brain injury after ICH^8^,^9^.

The P2X7 receptor, previously known for its cytotoxic activity, is an ATP-gated, non-selective cation channel which belongs to the family of ionotropic P2X receptors^10^,^11^. Evolving evidences indicate that P2X7 receptor plays a pivotal role in central nervous system pathology; genetic deletion and pharmacological blockade of P2X7 receptor provide neuroprotections toward various neurological disorders, such as ischemic stroke, neurotrauma, epilepsy, neuropathic pain, multiple sclerosis, and Alzheimer’s disease^12^,^13^. Our previous studies also demonstrated that P2X7 receptor involved in the neuroinflammation and apoptosis after subarachnoid hemorrhage^14^,^15^.

However, it is unclear whether endogenous P2X7 receptor participates in the pathophysiology process, especially in BBB disruption, of the early brain injury after ICH. And recent studies indicate that Ras homolog gene family member A (RhoA) might be the downstream signal of P2X7 receptor involved in blood brain barrier integrity modulation^16^,^17^. Therefore, in the present study, we sought to investigate the potential role and mechanism of P2X7 receptor in the blood brain barrier integrity after ICH.

## Results

### Time course and spatial expression of endogenous P2X7 receptor in rat brain after ICH

**Figure 1:**
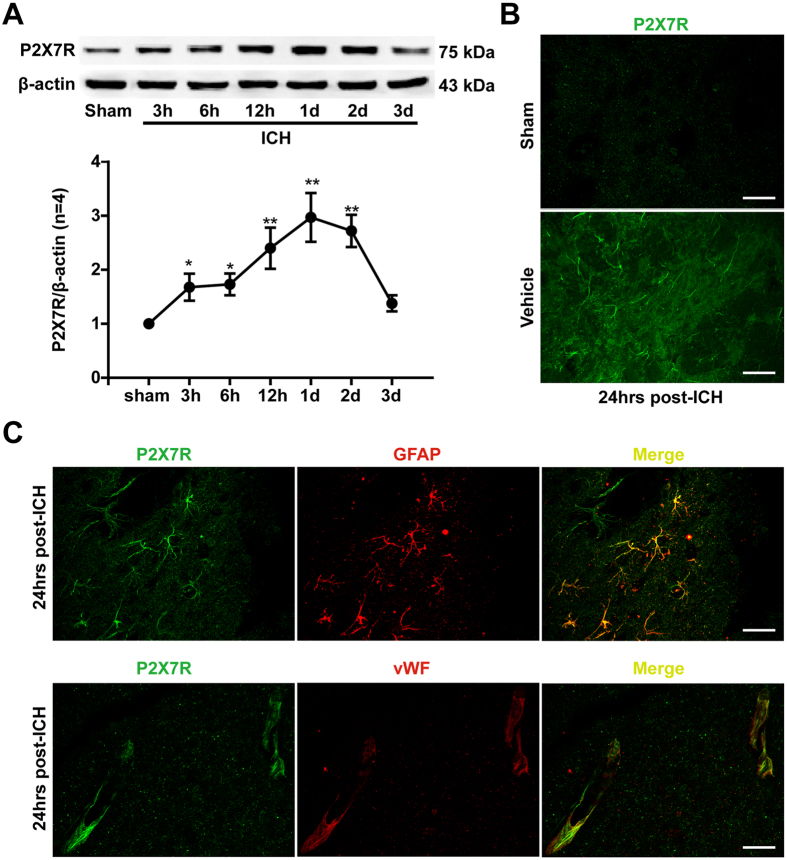
Time course and spatial expression of P2X7 receptor after ICH. (**A**) Representative bands and quantitative analyses of P2X7 receptor expression in the ipsilateral striatum. Relative densities of each protein have been normalized against the sham group. The cropped bands had been run under the same experimental conditions. (**B**) Representative immunofluorescence staining slices of P2X7 receptor (Green) in pericontusional striatum at 24 hours after ICH. Scalebar: 50 μm. (**C**) Representative immunofluorescence staining slices of P2X7 receptorwithglial fibrillary acidic protein (GFAP, red) and von Willebrand factor (vWF, red) in the perihematoma area at 24 hours following ICH. Triangle indicates hematoma area. Scale bar: 20 μm. *p < 0.05 vs. Sham; **p < 0.01 vs. Sham. n = 4 per group.

Western blot results (Fig. 1A) revealed that the protein level of P2X7 receptor in ipsilateral/right hemisphere was increased 3 hours after ICH and reached a peak at 24 hours in which the P2X7 receptor level was almost 3 times more than sham rats (p < 0.01). After that, the level of P2X7 receptor declined and returned close to the level of sham rats at 72 hours after ICH. Immunohistochemistry also exhibited that the P2X7 receptor expression in peri-contusional striatum was upregulated at 24 hours after ICH compared with the sham group (Fig. 1B). Furthermore, double immunofluorescence staining revealed that the P2X7 receptor immunoreactivity was mainly found peri-hematoma astrocytes and endothelial cells in ipsilateral striatum, which were marked with GFAP and vWF, respectively (Fig. 1C).

### Effects of A438079 treatment on neurobehavioral function, neuronal cell death, brain edema, and blood brain barrier integrity at 24 and 72 hours after ICH

**Figure 2:**
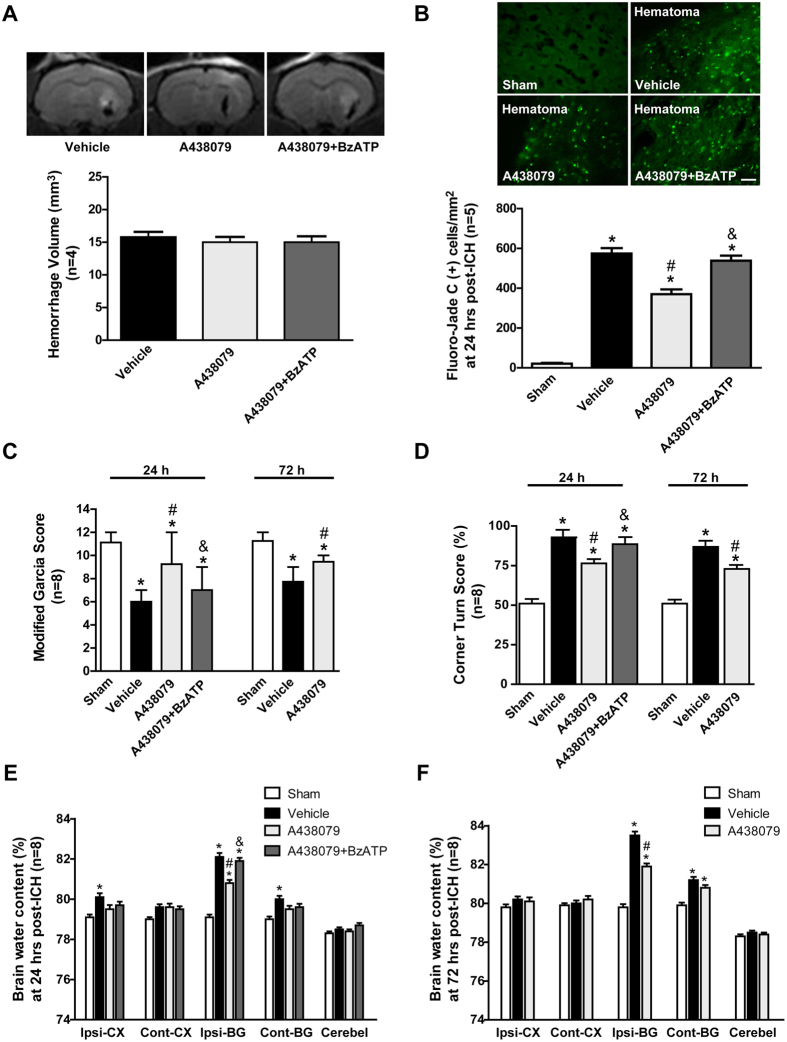
Effects of A438079 administration with/without BzATP pretreatment on outcomes at 24 and 72 hours after ICH. (**A**) Representative T2-weighted images and quantitative analyses of hematoma size at 24 hours after ICH. (**B**) Representative Fluoro-Jade C staining images and quantitative analyses of Fluoro-Jade C positive neuron around hematoma at 24 hours after ICH. (**C**) Modified Garcia scores and (**D**) Corner turn score at 24 and 72 hours after ICH in each group. (**E**)Brain water content assessment at 24 hours after ICH (**F**) Brain water content assessment at 72 hours after ICH. Scale bar: 50μm. *p < 0.05 vs. Sham; ^#^p < 0.05 vs. Vehicle; ^&^p < 0.05 vs. A438079; n = 4 for T2-weighted images, n = 5 for Fluoro-Jade C staining, n = 8 for others. Ipsi-CX: ipsilateral cortex; Cont-CX: contralateral cortex; Ipsi-BG: ipsilateral basal ganglia; Cont-BG: contralateral basal ganglia; Cerebel: cerebellum.

The hematoma volume of ICH were illustrated by T2*-weighted MRI (Fig. 2A). The ipsilateral striatum was damaged at 24 hours after ICH, but there was no significant difference of hematoma size between the ICH+Vehicle group, ICH+A438079 group and ICH+A438079+ BzATP group (p > 0.05) (Fig. 2A). These results demonstrated that our ICH rat models were reproducible and consistent. Moreover, the effects of those drugs did not act via the expansion of hematoma after ICH^18^,^19^,^20^.

We employed the Fluoro-Jade C staining to evaluate the neuronal cell death around hematoma. The results indicated ICH induced a significant increase in Fluoro-Jade C positive neuron (p < 0.05 vs. Sham) (Fig. 2B), and selective P2X7R inhibitor A438079 decreased the level of neuronal death in the peri-hematoma area after ICH (p < 0.05 vs. ICH+Vehicle) (Fig. 2B). Compared with sham group, ICH rats exhibited significant neurobehavioral deficits at both modified Garcia test and corner turn test. Administration of A438079 significantly alleviated those neurobehavioral deficits at 24 hours after ICH (p < 0.05 vs. ICH+Vehicle) (Fig. 2C,D). At 72 hours after ICH, A438079 treatment also ameliorated neurofunctional deficits (p < 0.05 vs. ICH+Vehicle) (Fig. 2C,D).

At 24 hours after ICH, brain water content in the bilateral basal ganglia and ipsilateral cortex were significantly increased compared with sham group. A438079 treatment significantly reduced brain water content in ipsilateral basal ganglia (p < 0.05 vs. ICH+Vehicle), but not in contralateral basal ganglia and ipsilateral cortex (p > 0.05 vs. ICH+Vehicle). However, brain water content in contralateral basal ganglia and ipsilateral cortex also had no significantly differences compared to ICH+Vehicle group (p > 0.05) (Fig. 2E). At 72 hours after ICH, only brain water content in ipsilateral basal ganglia was increased after ICH (p < 0.05 vs. Sham) and significantly reduced by A438079 (p < 0.05 vs. ICH +vehicle) (Fig. 2F).

**Figure 3:**
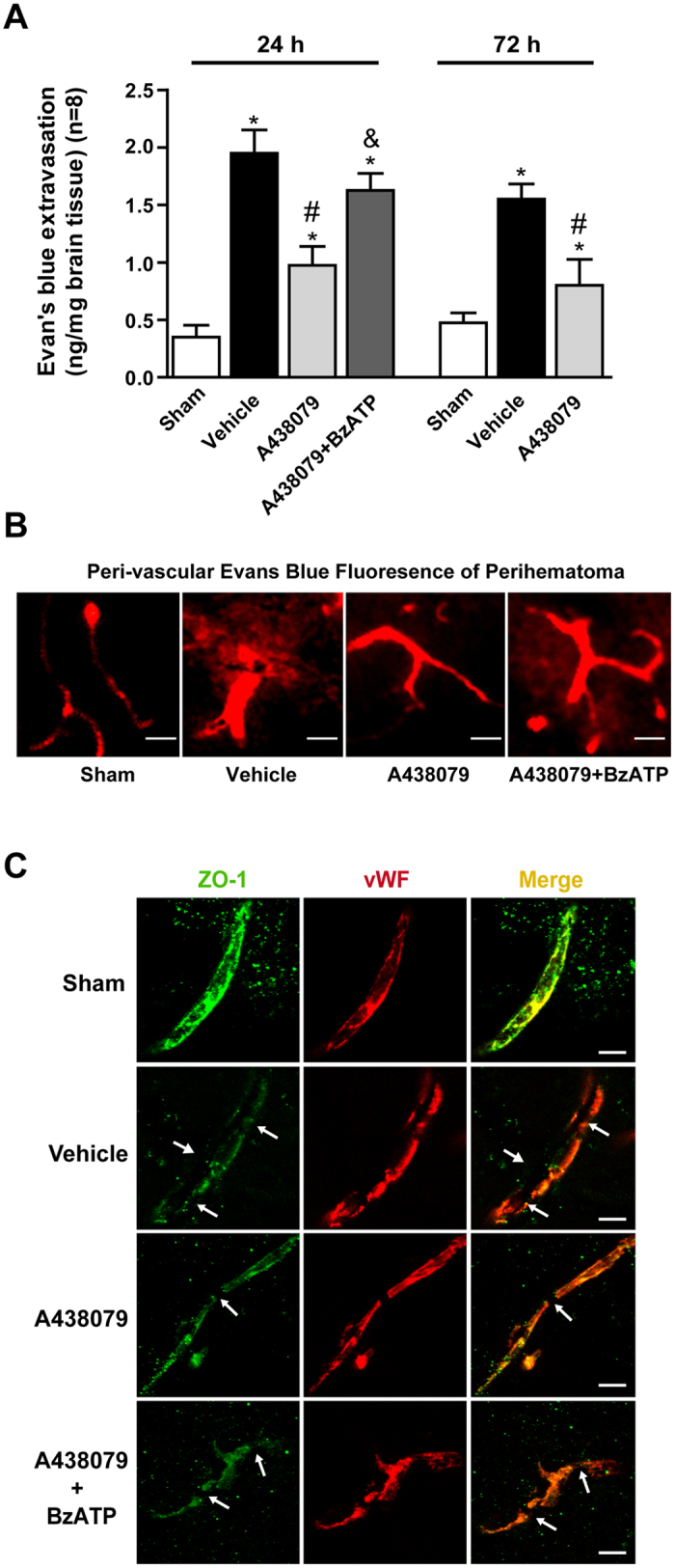
Effects of A438079 administration with/without BzATP pretreatment on blood brain barrier integrity at 24 and 72 hours after ICH. (**A**) Evans blue extravasation evaluation at 24 and 72 hours after ICH in each group. (**B**) Representative Evans blue fluorescence in ipsilateral striatum at 24 hours after ICH in each group. (**C**) Representative immunohistochemistry staining slices of zonula occluden-1 (ZO-1) and von Willebrand factor (vWF) at 24 hours after ICH. Arrow indicates the breakdown of continuous endothelia cell layer. *p < 0.05 vs. Sham; ^#^p < 0.05 vs. Vehicle; ^&^p < 0.05 vs. A438079. n = 8 per group.

Evans blue extravasation results showed that more extravagated dye was found in brain parenchyma at 24 and 72 hours after ICH (p < 0.05 vs. Sham) (Fig. 3A). Treated with A438079 significantly reduced Evans blue dye leakage (p < 0.05 vs. ICH+Vehicle). In addition, Evans blue fluorescence also exhibited that more Evans blue dye leakage around arteriole, and A428079 treatment reduced peri-vascular Evans blue dye (Fig. 3B). Furthermore, immunohistochemical staining showed that the structures of continuous endothelial cells (vWF) and ZO-1 in sham rats were broke up at 24 hours after ICH, and A438079 treatment effectively reduced those damages (Fig. 3C).

### Pretreated with BzATP abolished the protective effects of A438079 at 24 hours after ICH

Pre-ICH administration of BzATP significantly reversed the neuroprotective effects of A438079 on modified Garcia test, corner turn test, (p < 0.05 vs. ICH+A438079) (Fig. 2A,B). Also BzATP reversed the neuroprotective effects of A438079 on neuronal death (p < 0.05 vs. ICH+A438079) (Fig. 2C,D). In addition, BzATP pretreatment significantly increased post-ICH brain water content in the ipsilateral basal ganglia compared with ICH+A438079 group (p < 0.05) (Fig. 2E). Furthermore, BzATP aggravated Evans blue extravasation compared with ICH+A438079 group (p < 0.05) (Fig. 3A).

### Effects of P2X7 receptor knockdown on neurobehavioral functions and Evans blue extravasation at 24 hours after ICH

**Figure 4:**
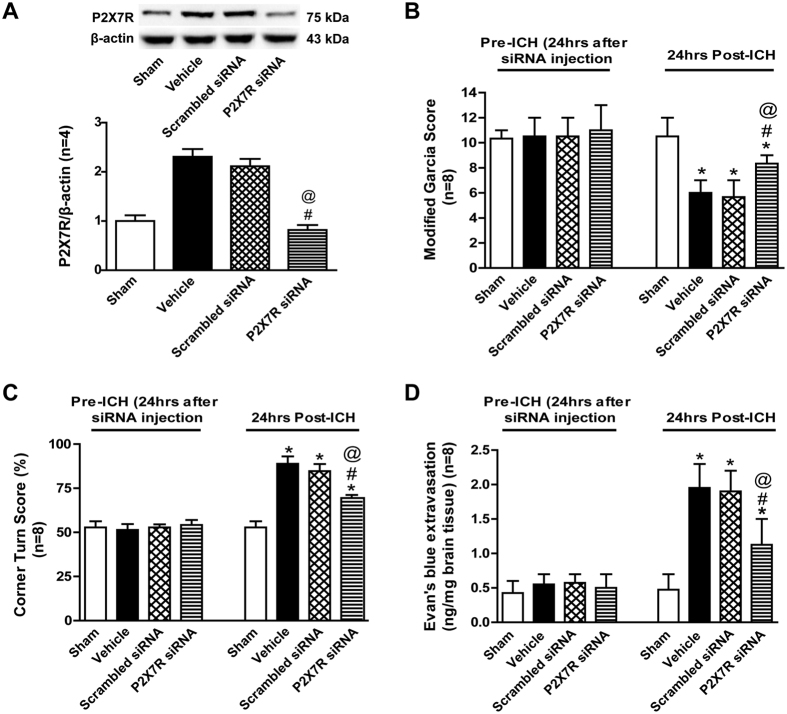
Effects of P2X7 receptor siRNA on outcomes at 24 hours after ICH. (**A**) Representative bands and quantitative analysis of the P2X7 receptor siRNA inhibiting effect. Relative densities of each protein have been normalized against the sham group. The cropped bands had been run under the same experimental conditions. (**B**)Modified Garcia scores and (**C**) Corner turn score at 24 hours after ICH in each group. (**D**) Evans blue extravasation evaluation at 24 hours after ICH in each group. *p < 0.05 vs Sham; ^#^p < 0.05 vs Vehicle; ^@^p < 0.05 vs Scramble siRNA. n = 8 per group. ICV = intracerebroventricular infusion.

The expression of P2X7 receptor was measured by western blot and no difference was observed in scrambled siRNA pretreatment group when compared with ICH+Vehicle group (p > 0.05), but P2X7 receptor siRNA pretreatment significantly inhibited the protein level of P2X7 receptor in ipsilateral striatum at 24 hours after ICH (p < 0.05 vs. ICH+Vehicle) (Fig. 4A).

There were no significant differences of neurobehavioral function and Evans blue extravasation among the Sham, ICH+Vehicle, ICH+Scrambled siRNA and ICH+P2X7 receptor siRNA groups (p > 0.05) (Fig. 4B–D). However, both vehicle- and scramble siRNA-pretreated ICH rats developed significant neurological deficits compared to sham group at 24 hours after ICH (p < 0.05), and no significant difference was seen between two groups (p > 0.05) (Fig. 4B,C). Similar to A438079, P2X7 receptor siRNA pretreated rats showed better neurobehavioral function during modified Garcia test (p < 0.05 vs. ICH+Vehicle or ICH+Scrambled siRNA) (Fig. 4B) and corner turn test (p < 0.05 vs. ICH+Vehicle or ICH+Scrambled siRNA) (Fig. 4C). In addition, P2X7R siRNA pretreated rats showed significant reducing Evans blue dye extravasation (p < 0.05 vs. ICH+Vehicle or ICH+Scramble siRNA) (Fig. 4D).

### Effects of P2X7 receptor modulating on RhoA activity and endothelial junction proteins expressions in ipsilateral striatum at 24 hours after ICH

**Figure 5:**
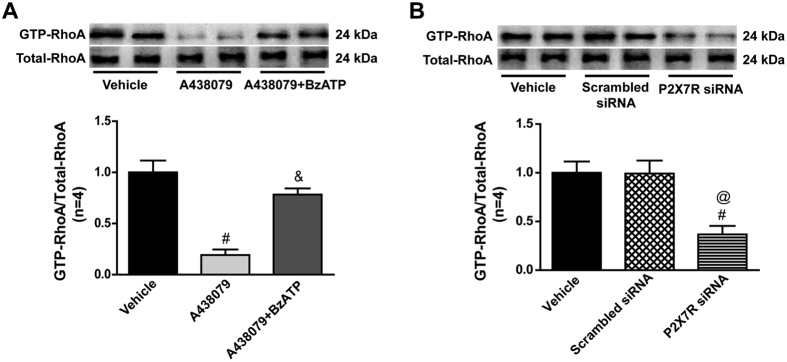
Effects of P2X7 receptor modulation on RhoA activity at 24 hours after ICH. (**A**) Representative bands and quantitative analysis of GTP-RhoA/Total-RhoA ratio after A438079 treatment with/without BzATP pretreatment at 24 hours after ICH in each group. (**B**) Representative bands and quantitative analysis of GTP-RhoA/Total-RhoA ratio P2X7 siRNA pretreatment at 24 hours after ICH in each group. Relative densities of each protein have been normalized against the vehicle group. The cropped bands had been run under the same experimental conditions. *p < 0.05 vs Sham; ^#^p < 0.05 vs Vehicle; ^&^p < 0.05 vs A438079; ^@^p < 0.05 vs Scramble siRNA. n = 4 per group.

The expression of activated RhoA (GTP–RhoA) increased after ICH when compared with the Sham group and was significantly decreased after administration of A438079 (p < 0.05 vs. ICH+Vehicle), but administration of BzATP reversed the effect of A438079 on GTP-RhoA expression(p < 0.05 vs. ICH+A438079) (Fig. 5A). Similar to A438079, P2X7 receptor siRNA pretreatment also significantly reduced the GTP-RhoA expression (p < 0.05 vs. ICH+Vehicle or ICH+Scrambled siRNA), while scrambled siRNA did not have this effect (Fig. 5B).

**Figure 6:**
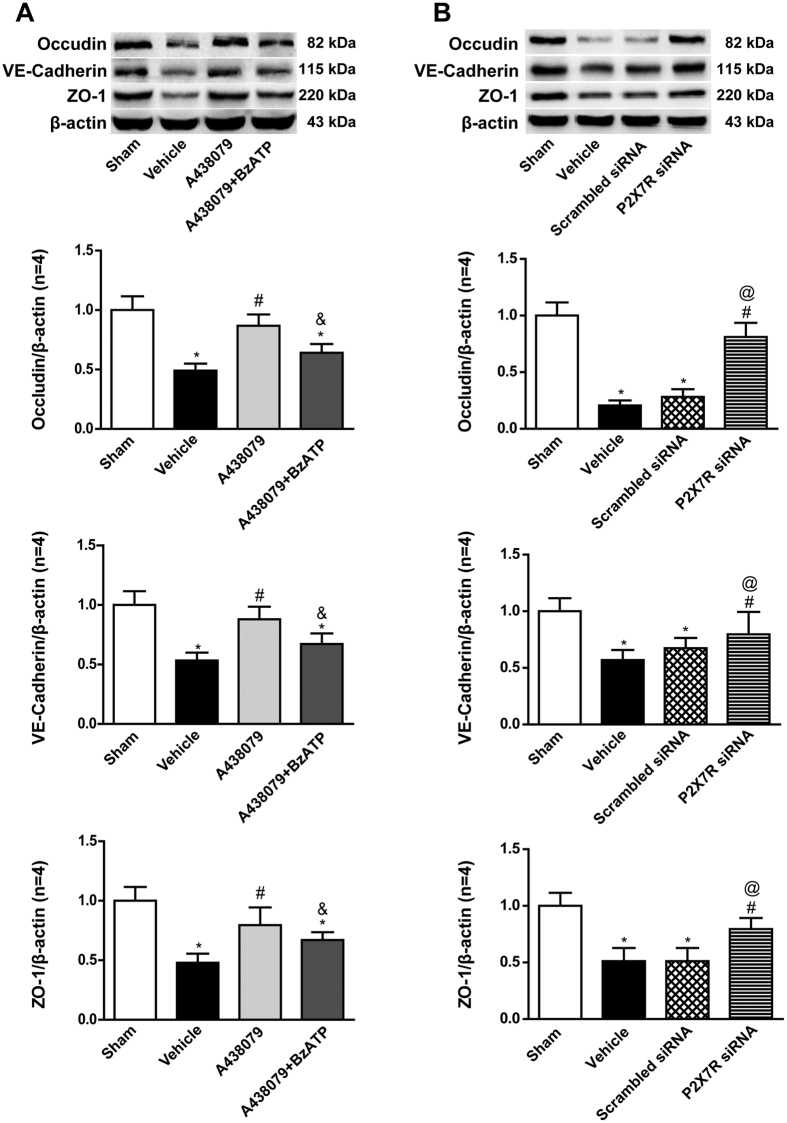
Effects of P2X7 receptor modulation on the expressions of endothelial junction proteins at 24 hours after ICH. (**A**) Representative bands and quantitative analysis of the expressions of Occudin, VE-Cadherin and zonula occluden-1 (ZO-1) after A438079 treatment with/without BzATP pretreatment at 24 hours post-ICH in each group. (**B**) Representative bands and quantitative analysis of the expressions of Occudin, VE-Cadherin and zonula occluden-1 (ZO-1) with P2X7 siRNA pretreatment at 24 hours after ICH in each group. Relative densities of each protein have been normalized against the sham group. The cropped bands had been run under the same experimental conditions. *p < 0.05 vs Sham; ^#^p < 0.05 vs Vehicle; ^&^p < 0.05 vs A438079; ^@^p < 0.05 vs Scramble siRNA. n = 4 per group.

Moreover, vehicle-treated ICH rats showed significantly decreasing expressions of Occludin, VE-Cadherin and ZO-1 (p < 0.05 vs. Sham). However, after administration of A438079, the expressions of Occludin, VE-Cadherin and ZO-1 were increased (p < 0.05 vs. ICH+Vehicle), and abolished by BzATP (p < 0.05 vs. ICH +A438079) (Fig. 6A). Immunofluorescence staining also demonstrated that A438079 preserved the continuous and linear pattern of ZO-1at 24 hours after ICH, but reversed by BzATP pretreatment (Fig. 3C). Similar to A438079, P2X7 receptor siRNA pretreatment also significantly increased the expressions of Occludin, VE-Cadherin and ZO-1 (p < 0.05 vs. ICH+Vehicle or ICH+Scrambled siRNA), while scrambled siRNA did not have this effect (Fig. 6B).

### RhoA inhibitor C3 transferase ameliorated brain injury after ICH through stabilization of endothelial junction proteins

**Figure 7:**
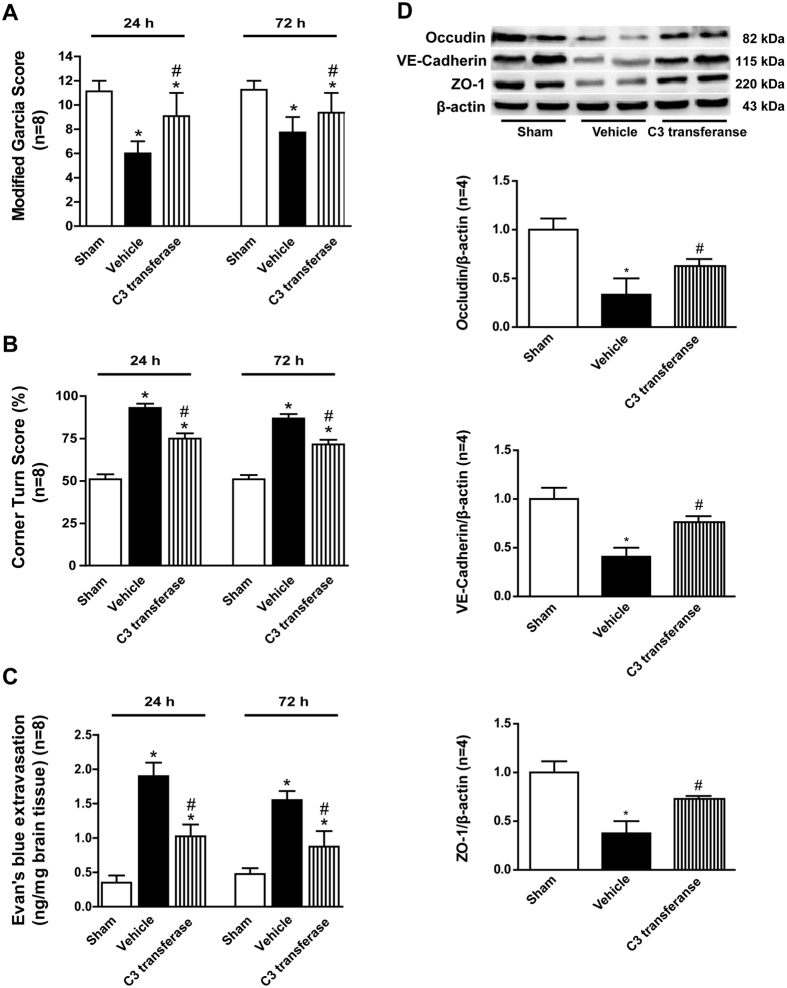
Effects of RhoA inhibitor C3 transferase on the outcomes and the expressions of endothelial junction proteins after ICH. (**A**) Modified Garcia scores and (**B**) Corner turn score at 24 and 72 hours after ICH in each group. (**C**) Evans blue extravasation evaluation at 24 and 72 hours after ICH in each group. (**D**) Representative bands and quantitative analysis of the expressions of Occudin, VE-Cadherin and ZO-1 after C3 transferase treatment at 24 hours post-ICH in each group. The cropped bands had been run under the same experimental conditions. *p < 0.05 vs Sham; ^#^p < 0.05 vs Vehicle; n = 4 per group.

C3 transferase significantly improved neurofunctional deficits (Fig. 7A,B) after ICH, in both the Garcia test and Corner turn test (p < 0.05 vs. ICH + Vehicle). Furthermore, a reduction in extravasated Evans blue dye (Fig. 7C) was found in the C3 transferase treatment group (p < 0.05 vs. ICH + Vehicle). And the levels of endothelial junction proteins (ratios of Occludin, VE-Cadherin, and ZO-1/β-actin) (Fig. 7D) were found significantly increased versus vehicle rats (p < 0.05).

### Effects of P2X7 receptor modulating exhibited the similar effects in collagenase induced ICH model

**Figure 8:**
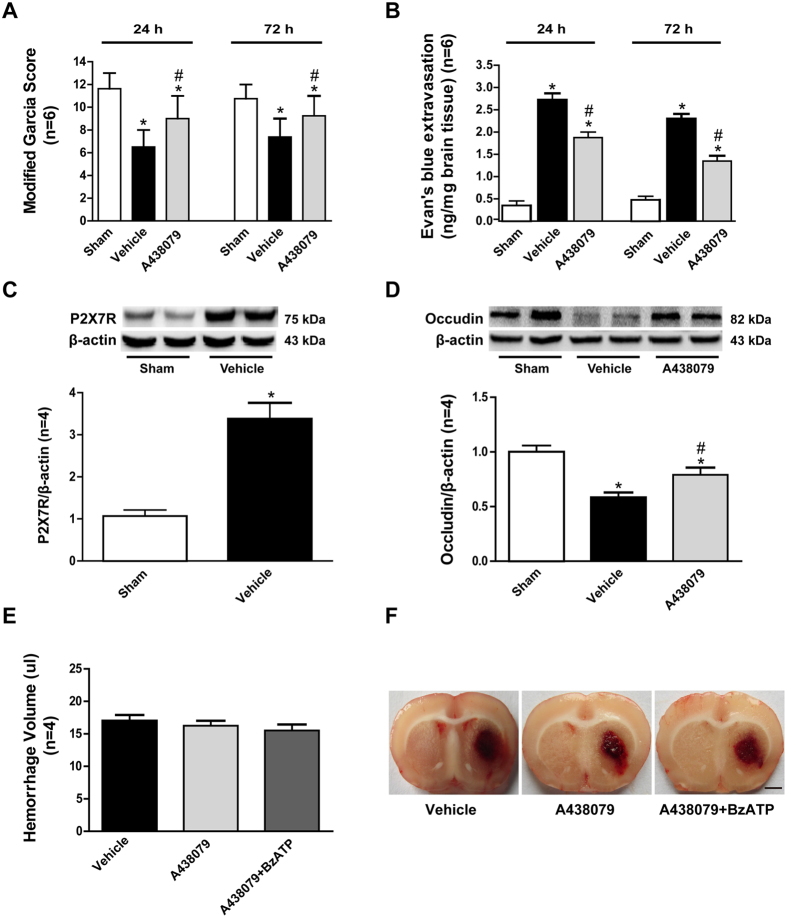
Effects of P2X7 receptor modulating exhibited the similar effects in collagenase induced ICH model. (**A**) Modified Garcia scores and (**B**) Corner turn score at 24 and 72 hours after ICH in each group. (**C**) Representative bands and quantitative analyses of P2X7 receptor expression in the ipsilateral striatum. Relative densities of each protein have been normalized against the sham group. (**D**) Representative bands and quantitative analysis of the expressions of Occudin at 24 hours post-ICH in each group. The cropped bands had been run under the same experimental conditions. (**E**) Quantitative analyses of hematoma size among each group and (**F**) representative coronal autopsy images at 24 hours after ICH. *p < 0.05 vs Sham; ^#^p < 0.05 vs Vehicle; n = 6 for modified Garcia score and Evan’s blue extravasation, n = 4 for others.

To verify the above results, we repeated some key experiments in collagenase induced ICH model at 24 hours after surgery. Similar to the data of autologous blood injection model, modified Garcia score significantly was decreased at 24 hours after collagenase induced ICH model, while Evan’s blue extravasation was increased at the same time (p < 0.05, vs. sham) (Fig. 8A,B). And P2X7R was significantly increased at 24 hours after collagenase induced ICH model (p < 0.05, vs. sham) (Fig. 8C). The selective P2X7R inhibitor A438079 significantly improved the modified Garcia score and reduced Evan’s blue extravasation (p < 0.05, vs. sham) (Fig. 8A,B). In addition, A438079 upregulated the expressions of Occudin comparing to the vehicle group (p < 0.05, vs. sham) (Fig. 8D).

The hematoma volume of collagenase induced ICH were illustrated by autopsy. No significant difference of hematoma size among groups demonstrated the reproducible and consistent of our ICH rat models (p > 0.05) (Fig. 8E,F). Also, the effects of those drugs did not act via the expansion of hematoma after collagenase induced ICH^18^,^19^,^20^.

## Discussion

In the present study, we investigated the time-course and spatial expressions of P2X7 receptor, as well as its function and mechanism to preserve blood brain barrier disruption following ICH injury. We found that endogenous P2X7 receptor increased at 3 hours after ICH with a peak at 24 hours, then returned to normal level at 72 hours after ICH. They mainly expressed on the astrocytes and endothelial cells at 24 hours after ICH. Either selective P2X7 receptor antagonist A438079 or P2X7 receptor siRNA alleviated neurological deficits, neuronal cell death, brain edema, and blood brain barrier disruption after ICH, which were associated with RhoA activation and down-regulated endothelial tight junction proteins. However, pretreated with BzATP, a P2X7 receptor agonist abolished those neuroprotective effects of A438079, inhibited RhoA activation, and increased endothelial tight junction proteins’ expressions. Furthermore, RhoA inhibitor C3 transferase treatment attenuated early brain injury and preserved BBB integrity through protection of endothelial junction proteins at 24 hours after ICH. Taking together, these data were consistent with our hypothesis that P2X7 receptor suppression could preserve blood-brain barrier integrity after ICH at least partially through inhibiting RhoA activity.

The ionotropic purinergic P2X7 receptor is an ATP-gated cation channel, whose activation controlled by adenosine triphosphate, allows influx of Ca^2+^ and Na^+^ and concomitant efflux of K^+^^10^. P2X7 receptor was reported to express throughout various cell-types, especially in macrophage-like cells in the brain and dynamically changes in response to various neurological disorders^11^,^21^. Previous studies demonstrated that P2X7 receptor activation contributes to microglial activation, astrogliosis and neurodegeneration^21^,^22^. Targeting the activation of P2X7 receptor might ameliorate immune-mediated central nervous system disorders^23^,^24^, including NLRP3 inflammasome mediated neuroinflammation after ICH^25^. A recent study has reported that 3,4-Methylenedioxymethamphetamine induced BBB disruption is ameliorated by P2X7 receptor antagonist through anti-inflammatory pathway^16^. However, whether and how P2X7 receptor regulates BBB function in early brain injury after ICH has not been determined. In the present study, we showed that endogenous P2X7 receptor was increased and colocalized with astrocytes^26^ and endothelial cells^27^,^28^ after ICH, which was consistent with the indispensable role of astrocyte P2X7 receptor on ischemic injury^29^. And the observations of this study also consisted with previous study in other acute brain injuries^14^,^15^,^30^,^31^,^32^ that either inhibits or knockdown P2X7 receptor alleviated neurological deficits, brain edema, and blood brain barrier, and pretreated with P2X7 receptor agonist reversed these effects. Due to the hematoma expansion could be one of the reasons to the second brain injury after ICH^33^,^34^,^35^. We evaluated the hematoma volume to demonstrated that our ICH rat models were reproducible and consistent among each groups. Moreover, our results indicated that the effects of modulating P2X7 receptor did not act via the expansion of hematoma after ICH^18^,^19^,^20^.

RhoA is a small guanosine triphosphate-binding protein known to regulate the formation of focal adhesions and stress fibers in response to growth factors^36^. Activating RhoA, lead to activating Rho-associated kinases and thereby inducing the phosphorylation and degradation of endothelial junction proteins to increase blood brain barrier permeability^37^,^38^,^39^,^40^. Increased activity of Rho kinase contributes to hemoglobin-induced early disruption of the blood-brain barrier *in vivo* after the occurrence of intracerebral hemorrhage^26^, which is consistent with our findings in the present study. In primary astrocyte cultures, activation of the P2X7 receptor with BzATP induced ~2.5-fold activation of RhoA, which was inhibited by pre-incubation brilliant blue G, another common used selective P2X7 receptor antagonist, indicating that RhoA is activated in the signaling pathway downstream of P2X7 receptors^17^,^41^. In the present study, we showed that P2X7 receptor localized in astrocytes around hematoma. Inhibiting P2X7 receptor decreased activated RhoA and increased endothelial junction proteins expression at 24 hours after ICH, which were reversed by P2X7 receptor agonist BzATP. These evidences showed the involvement of the P2X7R/RhoA singling pathway in the development of early brain injury after ICH, thus presenting a possible therapeutic target.

However, the present study has several limitations. Firstly, we only evaluated the neuroprotective effects of P2X7 suppression within 3 days after ICH. The long term outcome of ICH should be addressed to study the role of P2X7 receptor at normal protein level. Secondly, blockade of P2X7 receptor also exhibit anti-inflammatory effects^2^,^23^. Hence, we cannot eliminate the possibility that the anti-inflammatory or other effects also play a role in the neuroprotective effects of P2X7 receptor suppression. Furthermore, as thrombin is the most known factor leading to activation of RhoA and inhibition of RhoA prevented thrombin-induced endothelial permeability^26^, P2X7 receptor suppression might inhibit RhoA and preserve blood brain barrier integrity indirectly via affecting thrombin related signals and other pathways. Future studies are needed to clarify the specific mechanism of how P2X7 receptor regulates RhoA activity and endothelial junction proteins.

In summary, our findings provide a novel mechanism that inhibition of P2X7 receptor could alleviate neurological deficits, reduce brain edema, and prevent ICH-induced blood brain barrier permeability via inhibiting RhoA signal after ICH. And P2X7 receptor suppression treatment might be a promising strategy for ICH patients, which needs to be further explored.

## Methods

### Animals

Three hundred and forty (392) adult male Sprague-Dawley rats weighing 290–340 g (Experimental Animal Center of Third Military Medical University, Chongqing, China) were used in the present study. All experimental protocols were approved by the Ethic Committee of Southwest Hospital, and performed in accordance with the guidelines by the National Institutes of Health Guide for the Care and Use of Laboratory Animals. Rats were housed in a temperature- and humidity-controlled environment with food and water ad libitum in a 12-hour light/dark cycle and were acclimatized for more than 1 week before surgical procedures.

### Experimental Design

The experiments of the present study were designed as follows (see Supplemental Fig. 1).

### Experiment I

To determine the time course and spatial expressions of P2X7 receptor after ICH, 32 rats were randomly assigned into following groups, including: Sham (n = 6), ICH 3 hours (n = 4), ICH 6 hours (n = 4), ICH 12 hours (n = 4), ICH 24 hours (n = 6), ICH 48 hours (n = 4), and ICH 72 hours (n = 4). Western blots were used to detect the protein expressions of P2X7 receptor in ipsilateral/right hemisphere of each group (n = 4). The co-localization of P2X7 receptor with glial firillary acidic protein (GFAP) and von Willebrand factor (vWF) were examined respectively by double-labeling immunofluorescence at 24 hours after ICH (n = 2).

### Experiment II

To evaluate the effects of blockading P2X7 receptor, 204 rats were randomly divided into 4 groups, including Sham (n = 55), ICH+Vehicle (n = 55), ICH+A438079 (n = 55), ICH+A438079+BzATP (n = 33). Fluoro-Jade C staining (n = 5) was performed at 24 hours after ICH in ICH+Vehicle, ICH+A438079 and ICH+A438079+BzATP groups. Magnetic resonance imaging for hematoma size (n = 4), Modified Garcia test (n = 8), corner turn test (n = 8), brain water content (n = 8), Evans blue extravasation (n = 8) and perivascular fluorescence (n = 2) were assessed at 24 hours after ICH in all groups, and evaluated at 72 hours after ICH in Sham, SAH+Vehicle and SAH+A438079 groups. Double immunohistochemistry staining (n = 2) of ZO-1 and vWF were also performed in all groups at 24 hours after ICH.

### Experiment III

To evaluate the effects of P2X7 receptor knockdown, 40 rats were randomly divided into ICH+Scramble small interfering RNA (n = 20), and ICH+P2X7 receptor small interfering RNA (n = 20) groups. The data of Sham (n = 20) and ICH+Vehicle (n = 20) groups were shared with Experimental II. Western blot (n = 4), modified Garcia test (n = 8), corner turn test (n = 8), and Evans blue extravasation (n = 8) were conducted before ICH surgery and at 24 hours after ICH in all groups.

### Experiment IV

For mechanism study, 16 rats were divided into 4 groups: Sham (n = 4), ICH+Vehicle (n = 4), ICH+A438079 (n = 4), ICH+A438079+BzATP (n = 4). RhoA activation and protein levels of Occludin, VE-Cadherin, and ZO-1 in ipsilateral/right striatum were detected by RhoA activity assay and western blots at 24 hours after ICH in all groups.

Then 8 rats were assigned into 2 groups, including ICH+Scramble small interfering RNA (n = 4) and ICH+P2X7 receptor small interfering RNA (n = 4) groups. The data of Sham (n = 4) and ICH+Vehicle (n = 4) groups were shared with the experiment above. All four groups were conducted to evaluate the Rho activation and the expressions of Occludin, VE-Cadherin and ZO-1 in ipsilateral/right striatum at 24 hours after ICH.

### Experiment V

In order to validate activated RhoA could disintegrate endothelial junction proteins and destroy BBB integrity in the ICH model, 60 rats were divided into 3 groups: Sham (n = 20), ICH+Vehicle (n = 20), ICH+C3 transferase (n = 20). Modified Garcia test (n = 8), Corner Turn test (n = 8), Evans blue extravasation (n = 8) were assessed at 24 and 72 hours after ICH. Western blots of endothelial junction proteins of Occludin, VE-Cadherin, ZO-1 in ipsilateral/right striatum were conducted at 24 hours after ICH in all groups (n = 4).

### Experiment VI

To verify the results in autologous blood injection ICH model, 52 rats were randomly divided into 4 groups: Sham (n = 16), ICH+Vehicle (n = 16), ICH+A438079 (n = 16), ICH+A438079+BzATP (n = 4), and repeated key experiments in collagenase induced ICH model, including: modified Garcia score and Evans’s blue extravasation at 24 and 72 hours after ICH (n = 6); P2X7 receptor and Occludin expressions at 24 hours after ICH (n = 4); The hemorrhage volume at 24 hours after ICH (n = 4).

### ICH Model

ICH model was induced by autologous blood stereotaxic injection as our previous work^42^. Rats were anesthetized intraperitoneally with sodium pentobarbital (50 mg/kg), then positioned in a stereotaxic frame(Xi’an Northwest Optical Instrument Factory, Xi’an, China), and a cranial burr hole (1 mm) was drilled on the right coronal suture 3.5 mm lateral to the midline. Then, 50 ul autologous blood, taken from the right femoral artery, were infused into the right striatum within 60 seconds through a 30-gauge needle according to the following coordinates relative to bregma: 0.2 mm anterior, 5.5 mm ventral, and 3.5 mm lateral. The needle was kept in position for an additional 10 min to prevent backflow and the craniotomies were sealed with bone wax later. During surgical procedure, rats were kept at approximately 37 °C on an electric heating blanket, and then housed separately until complete recovery from anesthesia. Sham-operated rats underwent the same procedure except sterile saline injection instead of autologous blood.

The key results were verified in collagenase induced ICH model. Briefly, rats were anesthetized and positioned prone in a stereotactic head frame (Kopf Instruments, Tujunga, CA). A scalp incision was made along the midline and a burr hole (1 mm) was drilled on the right side of the skull at coordinates of 3 mm lateral to bregma and 5 mm ventral to the cortical surface. 1 μl (0.2 μl/min) saline containing 0.2 units of bacterial collagenase (type VII; Sigma Aldrich, St. Louis, MO) was injected stereotaxically into the striatum by using a Nanomite Syringe Pump (Harvard Apparatus, Holliston, MA). After injection, the Hamilton syringe was left in place for 5 minutes. The needle was slowly removed in additional 5 minutes to prevent backflow. The hole was sealed with bone wax and the wound was sutured.

### Post-surgical monitoring and sacrificing

Buprenorphine was used depending on the degree of observed distress or pain, and given for 6 hours-1 day depending on signs of pain or distress. The first administration of buprenorphine (0.02 mg/kg) was injected subcutaneously before anesthetic recovery and rats were observed for any sign of pain or distress. After surgical procedures, rats were held in clear cages with free access to rat chow and filtered water. And surgeons will check their general condition at least four times per day. At the specific time as experimental design, rats were anesthetized with sodium pentobarbital (50 mg/kg, intraperitoneally), and then were decapitated by guillotine. Brain specimen were removed carefully and conducted to the following experiments.

### Intracerebroventricular Infusion

Intracerebroventricular drug injection was performed as our previous study^43^. Briefly, rats were anesthetized with a pentobarbital (50 mg/kg) intraperitoneal injection and then fixed onto a stereotaxic head apparatus. The 26-gauge needle of a 10-μL Hamilton syringe (Microliter 701; Hamilton Company, Reno, NV) was inserted into the left lateral ventricle through a cranial burr hole at the following coordinates relative to bregma: 1.5 mm posterior; 1.0 mm lateral; 3.2 mm below the horizontal plane of bregma. Then drugs were infused by a microinfusion pump (Harvard Apparatus, Holliston, MA) at a rate of 0.5 μl/min. The needle was left in place for an additional 15 minutes after the infusion finishing, and then the incision was closed with sutures.

### Drug administration

A438079 (30 mg/kg, Tocris Bioscience, Bristol, United Kingdom)^16^ was dissolved in sterile saline and intraperitoneally injected at 0.5 hours after ICH. For 72 hours’ study, A438079 was administered three times at 0.5, 24, and 48 hours after ICH. BzATP (50 μg/rat in 5 μl sterile saline, Sigma Aldrich, St. Louis, MO)^15^ was intracerebroventricularly administered at 1 hour before ICH surgery.

In order to enhance the gene silence efficacy, two different P2X7 receptor siRNA (Sigma-Aldrich, St. Louis, MO) were mixed:

1. sense, 5′-CAGUGAAUGAGUACUACUA-3′ antisense, 5′-UAGUAGUACUCAUUCACUG-3′
2. sense, 5′-CUCUUGAGGAGCGCCGAAA-3′ antisense, 5′-UUUCGGCGCUCCUCAAGAG-3′

The scrambled siRNA (Dharmacon, Lafayette, CO) was used as the control. 500 pmol P2X7 receptor siRNA or scrambled siRNA, diluted with Lipofectamine 2000 (Life Technologies, Shanghai, China) according to the manufacturer’s protocol^44^, was injected intracerebroventricularly at 24 hours before ICH.

The selective RhoA inhibitor C3 transferase (40 ng/rat, Cytoskeleton, Denver, CO)^26^ was dissolved in water and independently administered into hematoma 30 minutes after ICH.

### Magnetic Resonance Imaging

T2-weighted image was performed as previous^45^ to evaluate the hematoma size in the ICH+Vehicle, ICH+A438079 and ICH+A438079+BzATP groups at 24 hours after autologous blood induced ICH. The rats were anesthetized with isoflurane at 2% in ambient air during each imaging procedure. Contiguous coronal slices of hematoma were imaged with a resolution matrix = 256 X 256, field of view (FOV) = 35 mm × 35 mm, slice number = 15, slice thickness = 0.5 mm, slice gap = 0, flip angle = 25, TR/TE = 4000 ms/60 ms and 200 ms/5 ms. A single technician ran all the image acquisition in a blinded manner. The hematoma volume measured on images was calculated by using an Image J software package (National Institutes of Health, Bethesda, MD).

### Modified Garcia Test

A 12-point score system named modified Garcia test was conducted in a blinded fashion as previous^46^,^47^, and used to evaluate neurological deficits at 24 and 72 hours after ICH. Briefly, this test consisted of 4 individual tests covering side stroke, vibrissae touch, limb symmetry, and lateral turning. A score of 0 (worst performance) to 3 (best performance) was given for each sub-test and a total Garcia score was calculated as the sum of all subtests.

### Corner Turn Test

Corner turn test was also conducted at 24 and 72 hours after ICH to evaluate the neurological deficits as previous^48^. Briefly, rats were allowed to walk into a 30-degree corner. When exited the corner, the rat could turn either to the left or the right, and this choice was recorded. Trials were repeated 10 times with 30 seconds interval, and the percentage of right turns was calculated.

### Brain Water Content

Brain water content was evaluated as previously reported^49^. Briefly, brain specimen was quickly divided into ipsilateral cortex (Ipsi-CX) and basal ganglia (Ipsi-BG) and contralateral cortex (Cont-CX) and basal ganglia (Cont-BG). All specimens were weighed as wet-weight, then dried in an oven at 105 °C for 72 hours and weighed again as dry-weight. Brain water content was calculated as (wet weight – dry weight)/wet weight × 100%.

### Evans Blue Extravasation and Fluorescence

Evans blue extravasation and fluorescence were performed as reported previously^43^. Briefly, the Evans blue dye (2%, 5 mL/kg; Aladdin, Shanghai, China) was injected and administered >2 minutes into the left femoral vein under anesthesia, where it was allowed to circulate for 60 minutes. Then rats were euthanized by an intracardial perfusion with sterile saline. Then brain samples were weighed, homogenized in sterile saline, and centrifuged at 15000 g for 30 minutes. After that, equal volume of trichloroaceticacid was added to the resultant supernatant. Those samples were then incubated overnight at 4 °C and centrifuged again at 15000 g for 30 minutes. The resultant supernatant was spectrophotometrically quantified for the extravasated Evans blue dye at 620 nm.

For Evans blue florescence, sterile saline was replaced by 4% paraformaldehyde after intracardial perfusion. Then brain specimen was removed to be prepared for coronal brain sections (10 μm) as same as immunohistochemistry staining. Then, the red auto-florescence of Evans blue dye was observed on the slides using excitation and emission fitter for red florescence^50^ (Olympus OX51, Tokyo, Japan).

### Western Blotting and Rho Activation Assay

Western blot was performed as reported previously^48^. After sacrificing, brain tissues around the lesion sites in right striatum were collected. Following primary antibodies were used: anti-P2X7 receptor (Millipore, Temecula, CA), anti–ZO-1 (Invitrogen, Grand Island, NY), anti-Occludin (Abcam, Cambridge, MA), and anti–VE-Cadherin (Santa Cruz Biotechnology, Santa Cruz, CA). β-actin was used as an internalloading control by using anti–β-actin primary antibody (Beyotime, Shanghai, China). GTP-RhoA and total-RhoA were detected by using Rho Activation Assay Kits (Millipore, Temecula, CA). Results were expressed as a relative density ratio, normalized to the mean value of the Sham group or ICH+Vehicle group.

### Immunohistochemistry Staining

Immunohistochemistry staining for brain was performed on fixed frozen section as previously described^43^, with primary antibodies: anti-P2X7 receptor (Millipore, Temecula, CA), anti-GFAP (Abcam, Cambridge, MA), anti–ZO-1 (Invitrogen, Grand Island, NY), and anti-vWF (Santa Cruz Biotechnology, Santa Cruz, CA), and followed by fluorescein isothiocyanate-conjugated and Texas Red-conjugated secondary antibodies (Jackson Immunoresearch, West Grove, PA). The colocalization of P2X7 receptor with GFAP or vWF and the colocalization of ZO-1 with vWF were examined by a confocal microscope (LSM780, Zeiss, Jena, Germany).

### Hematoma Volume

Hematoma volume of collagenase induced ICH model was analyzed by autopsy at 24 hours after ICH. After ICH rats were sacrificed, contiguous coronal slices of hematoma were prepared and the hematoma volume measured on images was calculated in a blinded manner by using Image J software package (National Institutes of Health, Bethesda, MD).

### Statistical Analysis

Data were expressed as mean ± SEM. Neurobehavioral data were analyzed by using Kruskal-Wallis One Way Analysis of Variance on Ranks, followed by the Student-Newan-Keuls Method. All other data were analyzed by using One Way Analysis of Variance followed by Tukey post hoc test. P value < 0.05 was considered statistically significant. All statistical analyses were performed by using SigmaPlot 10.0 for Windows (Systat Software Inc., San Jose, CA).

## Additional Information

**How to cite this article**: Zhao, H. *et al.* P2X7 Receptor Suppression Preserves Blood-Brain Barrier through Inhibiting RhoA Activation after Experimental Intracerebral Hemorrhage in Rats. *Sci. Rep.* **6**, 23286; doi: 10.1038/srep23286 (2016).

## Supplementary Material

Supplementary Information
