# Correction: P2X7 Receptor Suppression Preserves Blood-Brain Barrier through Inhibiting RhoA Activation after Experimental Intracerebral Hemorrhage in Rats

**DOI:** 10.1038/s41598-026-54497-x

**Published:** 2026-06-04

**Authors:** Hengli Zhao, Xuan Zhang, Zhiqiang Dai, Yang Feng, Qiang Li, John H. Zhang, Xin Liu, Yujie Chen, Hua Feng

**Affiliations:** 1 Department of Neurosurgery, Southwest Hospital, Third Military Medical University, Chongqing, China; 2 Department of Anesthesiology, Neurosurgery and Physiology, Loma Linda University, California, USA

Correction to: *Scientific reports* 10.1038/srep23286, published online 16 March 2016

This Article contains an error.

Due to an error during manuscript preparation, the number of animals listed in the first sentence of the Methods section under the subheading ‘Animals’ is incorrect.

As a result,

“Three hundred and forty (392) adult male Sprague-Dawley rats weighing 290–340 g (Experimental Animal Center of Third Military Medical University, Chongqing, China) were used in the present study.”

should read:

“Three hundred and forty (340) adult male Sprague-Dawley rats weighing 290–340 g (Experimental Animal Center of Third Military Medical University, Chongqing, China) were used in the present study.”

