# Supplementary material for: P2X7 Receptor Suppression Preserves Blood-Brain Barrier through Inhibiting RhoA Activation after Experimental Intracerebral Hemorrhage in Rats
Source: Sci Rep. 2016 Mar 16;6:23286. doi: 10.1038/srep23286 (PMC4793194; doi:10.1038/srep23286)
Supplement: Supplementary Information [file srep23286-s1.pdf]

P2X7 Receptor Suppression Preserves Blood-Brain Barrier through Inhibiting RhoA Activation after Experimental Intracerebral Hemorrhage in Rats

Hengli Zhao[1], Xuan Zhang[1], Zhiqiang Dai[1], Yang Feng[1], Qiang Li[1], John H. Zhang[2], Xin Liu[1], Yujie Chen[1,*], Hua Feng[1,*]

1 Department of Neurosurgery, Southwest Hospital, Third Military Medical University, Chongqing, China;

2 Department of Anesthesiology, Neurosurgery and Physiology, Loma Linda University, California, USA.

* Corresponding author:

Hua Feng, MD, PhD

Department of Neurosurgery, Southwest Hospital, Third Military Medical University. 29 Gaotanyan Street, Shapingba District, Chongqing, 400038, China.

E-mail: fenghua8888@vip.163.com

Phone: (86)023-68754153, Fax: (86)023-68763954

and

Yujie Chen, MD, PhD

Department of Neurosurgery, Southwest Hospital, Third Military Medical University, Chongqing, China. 29 Gaotanyan Street, Shapingba District,

Chongqing, 400038, China.

E-mail: yujiechen6886@foxmail.com

Phone: (86)023-68765265, Fax: (86)023-68765265

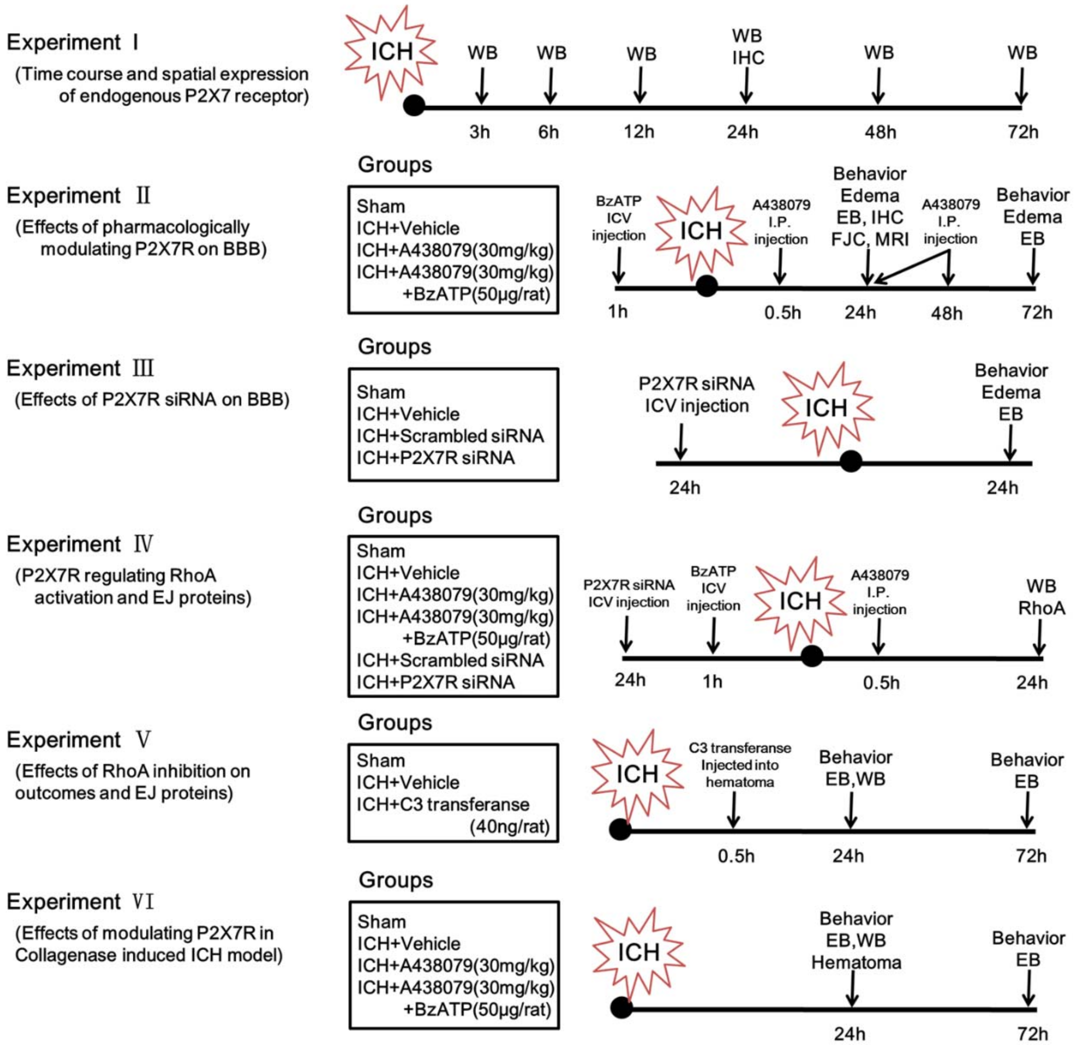

Supplemental Figure 1. Experimental design and animal groups' classification. ICH: Intracerebral hemorrhage; BBB: Blood brain barrier; P2X7: P2X7 receptor; EJ: endothelial junction; WB: Western blot; EB: Evans blue extravasation and fluorescence; FJC: Fluoro-Jade C staining; MRI: Magnetic Resonance Imaging; IHC: immunohistochemistry; ICV: intracerebralventricular; I.P.: intraperitoneal; Hematoma: Hematoma volume.
